# Genetic and Molecular Characterization of Flagellar Assembly in *Shewanella oneidensis*

**DOI:** 10.1371/journal.pone.0021479

**Published:** 2011-06-22

**Authors:** Lin Wu, Jixuan Wang, Peng Tang, Haijiang Chen, Haichun Gao

**Affiliations:** Institute of Microbiology and College of Life Sciences, Zhejiang University, Hangzhou, Zhejiang, China; University of Groningen, The Netherlands

## Abstract

*Shewanella oneidensis* is a highly motile organism by virtue of a polar flagellum. Unlike most flagellated bacteria, it contains only one major chromosome segment encoding the components of the flagellum with the exception of the motor proteins. In this region, three genes encode flagellinsaccording to the original genome annotation. However, we find that only *flaA* and *flaB* encode functional filament subunits. Although these two genesare under the control of different promoters, they are actively transcribed and subsequently translated, producing a considerable number of flagellin proteins. Additionally, both flagellins are able to interact with their chaperon FliS and are subjected to feedback regulation. Furthermore, FlaA and FlaB are glycosylated by a pathwayinvolving a major glycosylating enzyme,PseB, in spite of the lack of the majority of theconsensus glycosylation sites. In conclusion, flagellar assembly in *S. oneidensis* has novel features despite the conservation of homologous genes across taxa.

## Introduction

In environments where living conditions change constantly, microorganisms must respond to those changes rapidly. One of common approaches is that cells move away from detrimental environments and reach relatively favorable niches. Bacteria use a wide variety of cellular structures to facilitate motility, of which the flagellum is the most important and thus the best studied [1]. More than 80% of known bacterial species are motile by means of flagella [2]. In addition, flagella are indispensible in adhesion to substrates, biofilm formation, pathogenicity, and reduction of insoluble metal minerals [3]–[6]. In general, bacteria have either polar (*ie*. *Vibrio cholera*) or lateral (*ie*. *Escherichia coli*) flagella, which are alike structurally. Exceptions which have both are found [7]–[8]. Although the bacterial flagellum is one of the most complex of all prokaryotic organelles, the structure of the conventional flagellum is relatively well understood and excellent reviews are available [1], [9]–[10].

A flagellar system is tightly regulated because its synthesis and functioning is highly costly for the cell (about 2% of biosynthetic energy expenditure in *E. coli*) [10]–[11]. Genes encoding proteins involved in flagellum synthesis are organized into an ordered cascade in which the expression of genes at a given level is required for the expression of other genes at a lower level of assembly [10]. In Gram-negative bacteria, the regulatory cascades for lateral and polar flagella are dramatically different [12]. In bacteria with lateral flagella,at the top of the hierarchy is the *flhDC* operon, encoding the FlhD and FlhC proteins, which are essential for expression of downstream flagellar genes [12]. In the case of polar flagella, genes are transcribed in a four tiered hierarchy [13]. It is well established that a σ^54^-associated transcription activator is the master regulator at the top level among microorganisms in which such a master regulator has been identified [14]. This master regulator controls transcription of genes in the second tier, which encode components of the MS ring-switch complex as well as the regulatory factors FlrB, FlrC and FliA (σ^28^). FlrB and FlrC are responsible for transcription of genes in the third tier, most of which encode the basal body-hook, cap, and some of flagellins. The rest of flagellar genes encoding flagellins, the anti-sigma factor flgM, and the motor components, which make up the fourth tier, are σ^28^-dependent.

Owing to countless scientific efforts along with advances in glycoprotein detection and identification, it is now established that flagellins are heavily glycosylated. Studies on *C. jejuni* and *H. pylori* have illustrated two O-linked-glycosylation pathways, Pse and Leg [15]–[16]. Such modification via the *O*-glycan pathway is essential for flagellum assembly and bacterial motility [17]. However, *C. jejuni* 81–176 is the only strain for which the sites of glycosylation are known [18]. Although 19 serine or threonine residues, of which 18 reside in the D_2_–D_3_ domains, can be glycosylated only 8 are required for motility and flagellar assembly [19]–[20]. The D_2_–D_3_ domains are heavily glycosylated because they form the projections on the filament surface [21].

The *Shewanella* species have expanded during the last two decades as an important family of facultative Gram-negative anaerobes [21]. Initially, studies on this group of microorganisms were mostly aimed at exploring the ability for the bioremediation of metal/radionuclide contaminants in the environment. In recent years, *S. oneidensis*, the representative species of *Shewanellae*, has become a research model for respiration diversity, metabolic network, and biofilm formation [21]. For motility,all *Shewanella* species produce a polar flagellum. Since flagellar structures and gene organization are highly conserved, it is reasonable to assume that the synthesis of the flagella in *S. oneidensis* is similar to that in *V. cholera*, the paradigm for the polar flagellum and the only organism sharing extensive regions of similar gene order [9], [22]. Nevertheless, extensive diversity among flagellated bacteria exists in the content and organization of these flagellar genes and their regulation [23]–[25]. This may be especially true in the case of *S. oneidensis*, which has two sets of stator systems to drive flagellar rotation [26].

*Shewanella* species are extremely diverse in phenotypic or ecological features, making it difficult to accurately define the core characteristics of the genus [21], [27]. As one of the most genetically conserved organelles composed of a large number of components,flagella may perfectly serve that purpose [24]. Additionally, flagella of *Shewanella* species have been reported to be involved in formation of biofilms and pellicles [28]–[29]. In spite of the increasing importance placed on the organelle, it is surprising that flagella of *Shewanella* species have not been investigated in detail. In the present study we firstelucidated the content and organization of flagellar genes in *S. oneidensis* and compared these features to all sequenced *Shewanella* strains. We then systematically examined components with uncertain functions. We found that of the two major flagellin genesa major one was under the direct control of σ^28^and a minor one was also actively transcribed. A bacterial two-hybrid assay revealed that FliD was unable to interact with SO3234, the predicted chaperon of FliD, suggesting the lack of such a chaperon in the polarly flagellated bacteria. Furthermore, PseBwas identified to be essential forflagellinglycosylation although the entire glycosylation pathway remains elusive in *S. oneidensis*.

## Results

### *In silico* analysis of flagellar genesin *Shewanellae*

22 *Shewanella* genomes at Integrated Microbial Genomes at DOE Joint Genome Institute (http://img.jgi.doe.gov) were used for sequence comparison analyses. A preliminary analysis of flagellar genes revealed a high level of similarity, enabling us to use the best studied *S. oneidensis* as the representative species. Differences in other *Shewanella* genomes arediscussed in the text to provide a more comprehensive view of diversity.

**Figure 1:**
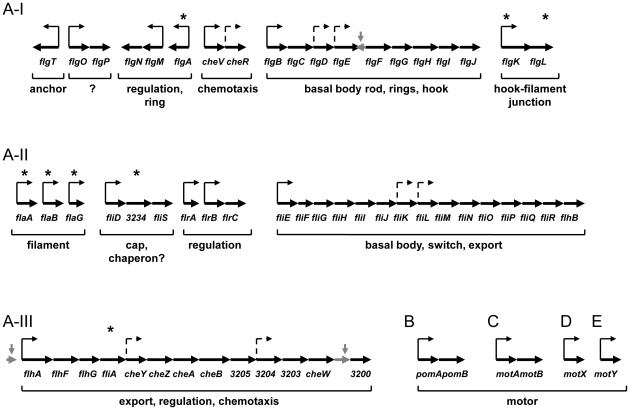
The *S. oneidensis* flagellar genes. The *S. oneidensis* genome containsone major regionfor flagellar genes (labelled A) as well as scattered motor genes (*pomAB* (*SO1529*-*30*), *motAB* (*SO4287*-*6*), *motX* (*SO3936*), and *motY* (*SO2754*). Region A, divided into three clusters I, II, III, encompassesgenes *SO3200-58*. Thick arrows denote ORFs (not drawn to scale) with corresponding genes below. Genes with an asterisk above were subjected to the physiological analyses in this study. Putative promoters are designated by thin arrows based on operon structures predicted at two sites (http://www.microbesonline.org; http://biocyc.org/SONE211586/). Solid and dash lines represent ones identified at both sites and ones found at only either site, respectively. Pseudogenes removed from the genome are in light grey.

*The S. oneidensis* genome contains up to70 geneson its chromosome encoding the flagellar and chemotaxis proteins that are homologous to those of other polar flagellar and chemotaxis systems [22]. These include the three sets of chemotaxis genes at different locations, of which only the third is likely to be functional since a mutation in *cheA-3*(*SO3207*) eliminated bacterial chemotactic response [30]. To elucidate organization of flagellar genes in *S. oneidensis*, flagellar gene systems from *E. coli* and *V. cholerae* were chosen as general references, representing the best understood and the closest in phylogeny. Unlike *E. coli* and *V. cholerae*, *S. oneidensis* allocates only one major location(*SO3200-58*) on the chromosome for flagellar genes and the third set of chemotaxis genes. This 59-gene fragment (approximately 155KB in length) can be divided into three clusters, resembling three regions of the polar flagellar genes of *V. cholerae* (Fig. 1). The structure of operons was derived from *in silico* analyses (http://www.microbesonline.org, http://biocyc.org/SONE211586/) with slight adjustments in reference to the well-defined counterparts of *V. cholerae* [13], [31]–[33]. In this 59-gene fragment, threemisidentified genes in the original annotation (*SO3246*, *SO3214*, and *SO3201* between *flgE* and *flgF*, *flhB* and *flhA*, and *cheW3* and *SO3200*, respectively) were removed when the genome was re-annotated, as presented at http://img.jgi.doe.gov.

The first cluster (A–I) includes*flgT*, *flgOP*, *flgNM*,*flgA*(*SO3253*), *cheV3-R3*, and *flgBCDEFGHIJK*(*SO3240*)*L*. While *flgM* and *cheVR* respectively encode an anti-σ^28^ factor and achemotaxis protein, the rest of the genes are annotated as structural genes for a sodium-driven motor ring (FlgT), a gene encoding a protein ofunknown function (FlgOP), one for a basal body rod, others for rings, and hook protein (FlgBCDEFGHIJKL), a chaperon (FlgN) and an assembly protein (FlgA) [34]–[35]. The *flgA* gene was not assigned in the original genome sequencing but it shows sufficient homology with *flgA* of other bacteria to be included so after validating its requirementfor motility [29], we designated it *flgA*. Another unassigned gene in the first annotation is *SO3240* (encoding an ortholog of FlgK), which was proposed to be non-functional due to two frameshifts in its predicted gene sequence. In contrast, all other sequenced *Shewanella* strains contain a full-length *flgK*, suggesting that there may be sequencing errors in the *flgK* gene of *S. oneidensis*.

The second cluster (A-II) of *S. oneidensis* flagellar genes consists of *flaA*(*SO3238*), *flaB*(*SO3237*), *flaG*, *fliD-SO3234-fliS*, *flrA*, *flrBC*, and *fliEFGHIJKLMNOPQR-flhB*. Except for *flrABC* encoding regulatory proteins, products of all these genes are components of the *S. oneidensis* flagellum such as filament, basal body, switch, and export proteins. Among genes in this cluster, *SO3237*, *SO3238* and *SO3234* were not named in the original annotation. Sequence analysis of SO3237 and SO3238 by BLAST revealed that the best fits were to flagellins, especially those of less than 300 a.a. in length. We therefore named *SO3237* and *SO3238flaB* and *flaA*, respectively. Previously, *flaA*, *flaB*, and *flaG* were proposed to encode flagellin subunits and removal of all three genes or *flaA* and *flaB* together resulted in a complete loss of motility [26], [36]. However, several lines of evidence suggest that FlaG is unlikely to be a flagellin subunit. These include that 1) the protein is too small (119 a.a.) to include all necessary domains of a functional flagellin [37], 2) the low sequence similarity to FlaA or FlaB (9%), 3) a *Pseudomonas fluorescensflaG* mutant was as motile as its parental strain [38], and 4) the *flaG* is the first gene of the four-gene “non-flagellin” operon *flaG*-*fliD*-*flaI*-*fliS* in *V. cholerae* [13].

A great diversity in flagellar filament genes among sequenced *Shewanella* strains was observed. Like *S. oneidensis*,12 other strains possess two genes encoding flagellins of 265–275 a.a., implying that flagellar filament subunits of this length represent the ancestral set of flagellins in *Shewanellae*. Intriguingly, *S. baltica* OS185 and OS195 possess four genes encoding flagellins while two other sequenced *S. baltica strains* (OS155 and 223) have only two (Fig. S1). The additional two flagellar filament genes reside on a fragment of 5.4 kb betweenanalogs of *S. oneidensisflaA* and *flaB*. These fragments appear to haveresulted from transposition events perhaps due to the presence of IS4 family transposase genes. The second largest group consists of 5 strains including *Shewanella* sp. MR-4, MR-7, *S. benthica*, *S. violacea*, and *S. frigidimarina*, whosegenes encode theflagellin subunits of 465–482 a.a.. It is worth noting that *S. benthica* KT99 not only carries transposase gene between the flagellin gene and *flaG* but also have two additional genes encoding 104 a.a. and 344 a.a. proteins in place of the counterpart of *S. oneidensisflaB*. Given that both proteins exhibit high levels of sequence similarity to known flagellins, we speculate that this unusual structure may be the result of sequencing errors. The other two strains *S. pealeana* and *S. piezotolerans* contain genes encoding flagellins of 393–394 a.a. and 434–463 a.a., respectively. It is interesting to note that *S. piezotolerans* is the only strain whose two flagellins differ from each other bymore than 5 a.a..

The only ORF in the A-IIcluster that could not be functionally assigned is *SO3234*. The geneencodes a small protein (106 a.a.)of unknown function. Although the proteins are highly conserved among sequenced *Shewanella* strains, its counterparts in other organisms have not been confidently identified. In *V. cholerae* and *Vibrio parahaemolyticus*, the gene at the same location is designated *flaI* but its product shares sequence identity of less than 20% with SO3234 [13].

The third cluster (A-III) contains the chemotaxis genes *cheY3Z3A3B3W3*, the export gene *flhA*, the regulatory genes *fliA* and *flhFG*, and the four ORFs encoding proteins ofunknown function. The *fliA* gene product (σ^28^) is a sigma factor specific for expression of some late flagellar genes encoding the filament proteins, motor proteins and other flagellar-related secreted proteins [1]. In polarly flagellated bacteria, *flhF* and *flhG* encode proteins controlling the number and location of the flagella.

The flagellar genes that are not on this 59-gene fragment include two sets of motor genes: *pomAB*(*SO1529*, *SO1530*) and *motAB* (*SO4287*, *SO4286*), and two motor auxiliary protein genes: *motX*(*SO3936*) and *motY*(*SO2754*). Interestingly, *S. oneidensis* is the onlystrain containingboth proton-driven PomAB and sodium-driven MotAB whereas in all other sequenced *Shewanella* strains MotAB is lacking. A recent study on *S. oneidensis* has demonstrated that bothmotor units were neededto power the flagellum simultaneously with assistance from MotXY [26], [39].

### Development of suitable complementation plasmids for flagellar genes

The bacterial flagellar system is relatively stable in evolution and up to 24 core genes are found in every microorganism possessing flagella [24]. While genomic analyses allow for the direct detection of genes encoding most flagellar components, it is still necessary to carry out mutational analyses on uncertain ones for functional validation.

In our previous studies, broad host range vectors pBBR1MCS-2 and pBBR1MCS-5 were used for complementation by introduction of the intact gene and/or its promoter region into the multiple cloning site (MCS) of either plasmid [40]–[42]. However, the majority of the flagellar gene operons are unusually large (the predicted largest is 13 kb in *S. oneidensis*), consisting of multiple genes with different function. As a result, a vectors such as pBBR1MCS is not suitable because: 1) mutation(s) may be introduced during amplification ofthe targeted gene and its promoter; 2) the other genes within the same operon may interfere with complementation, especially those encoding regulatory proteins. To circumvent such an obstacle, we constructed two plasmids: pHG101, a promoter-less plasmidfor genes next to their promoter and pHG102,hosting the *S. oneidensis arcA* promoter for genes some distance from their promoter. The *arcA*promoterwas chosen because the gene has been found to be expressed at the considerable level under both aerobic and anaerobic conditions [40], [43]. Th *is* promoter with an MCS was generated by PCR (primers available upon requestunless otherwise noted).

In this study, all mutants that displayed phenotypes distinguishable from their parental strains were subjected to complementation. Although motility of each strain on both swimming and swarming plates was examined in comparison with the wild type only swimming results are presented for there was no statistically significant difference between swimming and swarming unless otherwise noted. The results aresummarized in Table 1 anddiscussed in the text when necessary.

**Table 1:** Motility of *S. oneidensis* flagellar mutants^a^.

Strain	Plasmid^b^	Gene on Plasmid^c^	RC of Swarming^d^	RC of Swimming^e^
WT			1	1
WT	pHG101		1.01±0.04	0.98±0.03
WT	pHG102		0.99±0.04	1.03±0.04
Δ*flgK*	pHG101		0.04±0.02	0.07±0.03
Δ*flgK*	pHG101	*flgK*	1.05±0.04	1.04±0.04
Δ*flaB*	pHG101		0.34±0.06	0.40±0.03
Δ*flaB*	pHG101	*flaB*	1.11±0.05	1.46±0.05
Δ*flaA*			0.97±0.06	1.08±0.05
Δ*flaA*Δ*flaB*	pHG101		0.03±0.01	0.05±0.02
Δ*flaA*Δ*flaB*	pHG101	*flaB*	1.00±0.04	1.07±0.06
Δ*fliA*	pHG102		0.05±0.03	0.03±0.01
Δ*fliA*	pHG102	*fliA*	0.95±0.04	0.99±0.03
Δ*fliD*	pHG101		0.03±0.01	0.04±0.01
Δ*fliD*	pHG101	*fliD*	0.98±0.06	1.07±0.06
Δ*SO3234*	pHG102		0.64±0.07	0.57±0.07
Δ*SO3234*	pHG102	*SO3234*	0.95±0.08	1.01±0.06
Δ*pseB*	pHG101		0.06±0.02	0.05±0.02
Δ*pseB*	pHG101	*pseB*	1.03±0.06	0.99±0.06

a The motility – the distance from the culture spot to the edge of the haze of motility.

b The vector in the strain to provide the same genetic background.

c The designated vector borne gene in the strain for complementation.

d RC of swarming, the swarming motility of each strain is normalized to that of the wild type strain on the same agar plate.

e RC of swimming, the swimming motility of each strain is normalized to that of the wild type strain on the same agar plate.

### *S. oneidensis* contains an intact and functional FlgK

**Figure 2:**
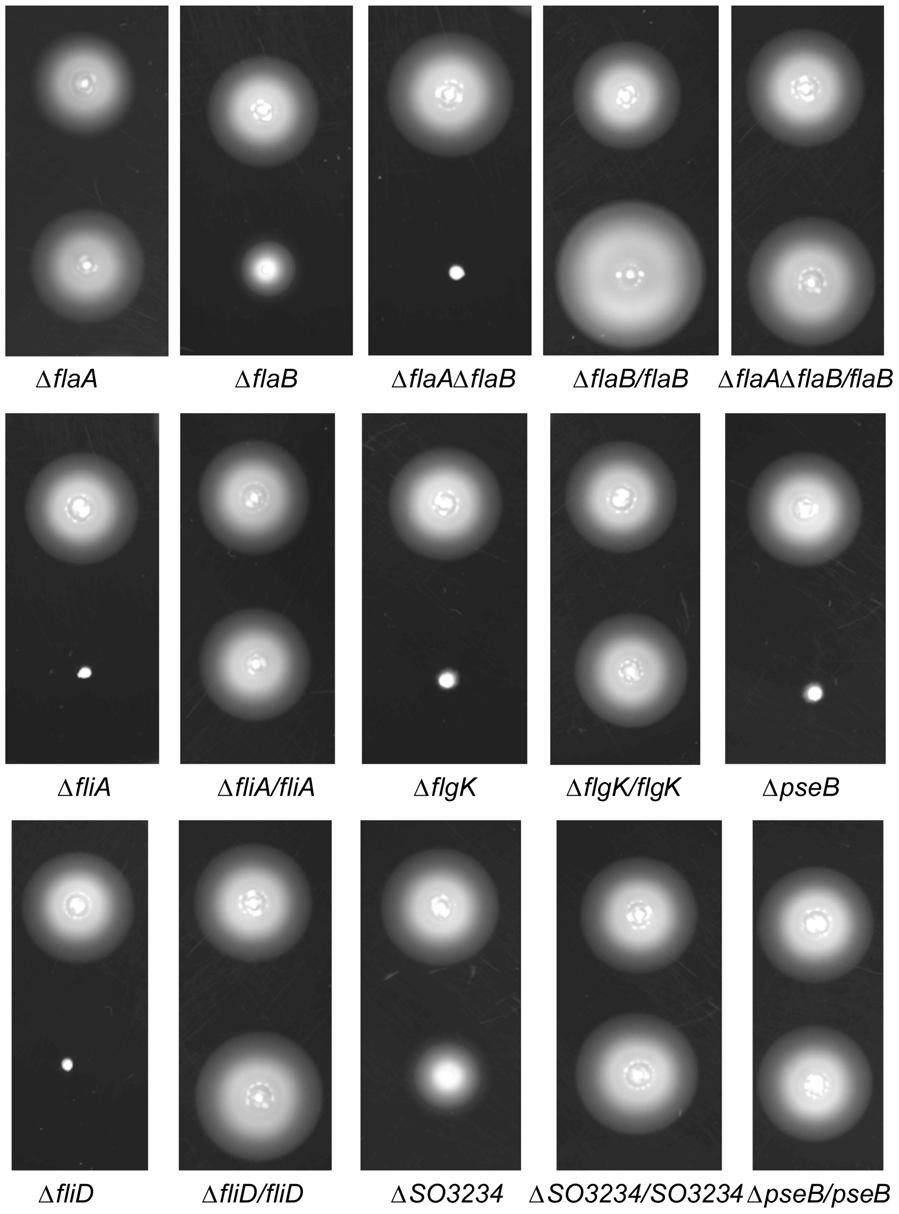
Motility of *S. oneidensis* wild type and isogenic mutation strains. In all panels, the mutant strain (lower) was compared to its parentalwild type (upper) in the swimming assay. Each mutant containing its complementation vector is presented as the mutant/the gene. For example, the Δ*flaB*/*flaB* strain refers to the Δ*flaB* mutant containing complementation vector pBBR-FLAB.

According to the genome sequence, two ORFs encoding proteins of 386 and 141 a.a. reside at the *flgK* locus. *flgK* encodes a hook-filament junction protein, which is essential for flagellar assembly and motility [11]. Given the synteny and the indispensible role of FlgK, it is unlikely that this crucial protein could be missing. To verify the sequence, an *S. oneidensis* culture with flagellated cells (confirmed by a microscope) was used as the chromosomal DNA source for gene amplification. Three independent sequencing results unambiguously indicated that *SO3240* in the original annotation missed out A and C immediately after nt1134 and nt1505, which introduced frame-shift mutations. Addition of these two nucleotides allows translation of the full length ORF, resulting in a protein of 641 a.a., which displayed sequence similarities (full length) more than 80% and 50% to FlgK in other sequenced *Shewanella* and *Vibrio* species, respectively. Based on this result, we named *SO3240* as *flgK*. To further validate the intactness of *SO3240*, we constructed an in-frame deletion strain. As expected, the mutant was non-motile, but the wild-type phenotype was restored when pHG101 carrying the gene was introduced into, confirming that FlgK is functional in *S. oneidensis* (Table 1) (Fig. 2).

### FlaB is the major flagellinsubunit for motility

In *Shewanellae*, genes encoding the flagellar filament subunits display the largest diversity asmentioned above, which warrants an in-depth analysis. Two flagellin genes are found in the *S. oneidensis* genome:*flaA*(*SO3238*)and *flaB*(*SO3237*). FlaA and FlaB share a sequence identity of 89% although they are rather short, only 272-3 a.a. in length compared to 376 a.a for *V. cholera*, 498 a.a. for *E. coli*, and 572 a.a. for *Campylobacter jejuni* [44]. Structural analyses on the 495 a.a. filament protein of *S. typhimurium* revealthat the protein consists of seven domains: D_0_-D_1_-D_2_-D_3_-D_2_-D_1_-D_0_, of which D_2_-D_3_-D_2_are not required for formation offull-length flagella for motility [37], [45]–[47]. We therefore speculate that the *S. oneidensis* subunits may retain only necessary domains. To test this hypothesis, alignments of the *S. oneidensis* FlaA and FlaB amino acid sequences with *S. typhimurium* LT2 FliC were performed. Indeed, the D_2_-D_3_-D_2_ domains were not found in either *S. oneidensis* flagellar filament subunit (Table 2).

**Table 2:** Alignment of *S. typhimurium* FliC with *S. oneidensis* FlaA and FlaB.

Domain	Residues within domain^a^		%identity with FliC^b^	
	FliC	FlaA/FlaB	FlaA	FlaB
D_0_ (N)	1–45	1–45	66	68 (91)^c^
D_1_ (N)	46–180	46–168	43	46 (86)
D_2_ (N)	181–190	–	–	–
D_3_	191–284	–	–	–
D_2_ (C)	285–407	–	–	–
D_1_ (C)	408–455	186–233	53	51(93)
D_0_ (C)	456–495	234–272	57	58 (94)

a The domain boundaries within the *S. oneidensis* FlaA and FlaB proteins were assigned based on alignment with *S. typhimurium* FliC.

b Per cent identity was determined using CLUSTALW with a gap penalty of 10.0 and a gap length penalty of 0.2.

c Numbers in bracket represent %identity between *S. oneidensis* FlaA and FlaB.

In *V. cholerae*, mutation of *flaA* completely abolished motility whereas the other four flagellin genes were dispensable [48]. *C. jejuniflaA* was essential for motility but a *flaB* deletion strain produced full-length functional flagellar filaments [49]–[50]. Moreover, in *V. parahaemolyticus*, loss of one of six flagellar filament genes had little or no effect on motility or flagellar structure [11]. Consequently, in *S. oneidensis* it is possible that FlaA or FlaB may not be essential for formation of full-length flagella despite the high degree of conservation in all of the domains of these two proteins.

To determine the role of these two genes, we constructed *S. oneidensis* strains containing an in-frame deletion in *flaA*, *flaB*, or *flaAflaB*. Motility assaysdemonstrated that compared to the wild type strain,the mutation in *flaA*had little or no effect on swimming motility but Δ*flaB* retained only about 40% of its swimming/swarming capabilitybased on the radius of the swimming rings (Table 1, Fig. 2). The double mutantΔ*flaA*Δ*flaB*, however, lost motility completely. To confirm the observation, Δ*flaB* wastransformed with the pHG101 containing *flgB*. Surprisingly, the strain with plasmid-borne *flgB* showed a stronger capacity for swimming than its parental wild type, which is likely due to multiple copies of the gene in the complemented strain. However, in the case of swarming, the motility increased much less significantly. This suggests that over-expressed *flaB*may elevate motility of individual cells as swarming motility is a form of movement in multicellular groups rather than as individuals [51]. To test whether over-expressed *flaB* alone is enough to promote motility, Δ*flaA*Δ*flaB* was complemented with *flaB*. A swimming assay showed that the plasmid borne *flaB* was only able to elevate motility to a level equal to that of the wild type. Examination of the Δ*flaB* cells by electron microscopy revealed that the *flaB* mutant produced heavily truncated flagella (data not shown). In contrast,Δ*flaA* displayed filament morphology similar to the wild type. All of these results indicate that both flagellin subunits are constituents of the filaments with FlaB dominating.

### *flaB* but not *flaA* is under the control of σ^28^

Our observation that one of the flagellins is predominantin the *S. oneidensis* flagellum is supported by other examples. In *V. cholerae*, transcription of the essential *flaA* was directed by σ^54^ whereas the other four non-essential flagellin genes were controlled by σ^28^ [48]. In contrast, in *C. jejuni* the essential flagellar filament gene *flaA* was under the control of σ^28^whereas the σ^54^-controlled *flaB* played a minor role [49]–[50]. Given the high degree of sequence identity between *S. oneidensis* FlaA and FlaB, the contrasting phenotypes of these two mutants are likely to bedue to differences in gene expression levels.

To elucidate the underlying mechanism determining the essentiality of FlaB in *S. oneidensis*, we first examined the promoter regions of *flaB* and *flaA*. The σ^28^–dependent promoters in the *S. oneidensis* genome have been predicted using pattern matching (PM) and iterative position specific score matrix (PSSM)-based approaches [52]. PM and PSSM identified σ^28^ binding sites in upstream regions of 9 and 12 genes, respectively, and only 6 were in common supporting the use of these programs. We then constructed the σ^28^ binding weight matrix using the experimentally verified σ^28^ binding sequences from *E. coli*, *S. typhimurium* and *V. cholerae* to screen for the σ^28^ binding sites in the *S. oneidensis* genome [13], [40], [43], [53]. In total, 169 putative σ^28^ binding sites were identified using RSAT with the default setting [54] as given in the supplemental material (Table S1). The σ^28^ recognition sites were found in the promoter regions of flagellar genes including *motY*, *flaB*, *flgM*, and *pomA*, all of which were among the top 15 most confident. Furthermore, these four sites are the only sites that have been identified by all three approaches (in our method, the top 15 were chosen for comparison). These results strongly suggest that *motY*, *flaB*, *flgM*, and *pomA* are under the control of σ^28^ in *S. oneidensis* in agreement with similar results in *V. cholerae* [13], [31].

**Figure 3:**
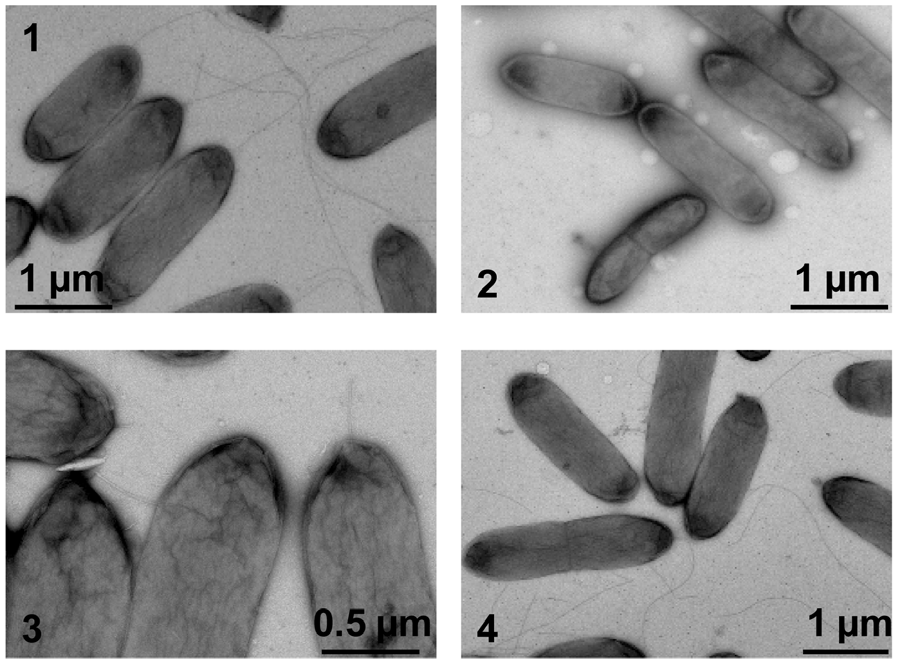
Transmission electron micrographs of *S. oneidensis* wild type and flagellar mutantstrains. Cells were stained with 1% phophotungstic acid and applied to TEM. 1. The wild type. 2.Δ*flgK*, aflagellated. 3. Δ*fliD*, truncated filaments. 4. Δ*SO3234*, flagella indistinguishable from that of the wild type.

**Figure 4:**
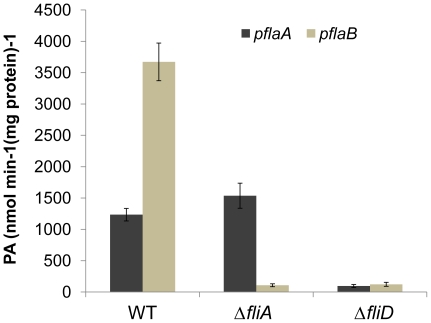
Promoter activities of *flaA* and *flaB* in *S. oneidensis* strains. Whole-cell lysates were prepared from *S. oneidensis* cultures in mid-exponential growth phase and assayed as described in Experimental Procedures. Promoter activities were determined by measuring β-galactosidase levels using *flaA*-*lacZ* and *flaB*-*lacZ* reporter constructs in the wild type, Δ*fliA*, and Δ*fliD* strains. Quantitation of the promoter activities was normalized to the total protein in each strain.

To experimentally validate that *flaB* is under the direct control of σ^28^, we fused each of the *flaA* and *flaB* promoters to the full-length *lac* operon in a newly developed reporter system [43]. For the promoter activity assay, a *fliA* null mutant was constructed to provide a genetic background lacking σ^28^. As expected, the mutant produced a truncated flagellum and lost motility completely, matching the phenotype of *fliA* defective mutants from various bacteria (Table 1, Fig. 2& 3). The reporter plasmids were introduced into the wild type and aΔ*fliA*strain and the activity of the *flaA* and *flaB* promoters was measured. Although both *flaA* and *flaB* were actively transcribed in the wild type background, the promoter activity of *flaB* was approximately three times higher than that of *flaA* (Fig. 4). In contrast, in the *fliA* defective background, *flaB* was barely transcribed whereas expression of *flaA* was not significantly altered. These results verify that *flaB* but not *flaA* is under the control of σ^28^.

### Both FlaA and FlaB are under the feedback control

**Figure 5:**
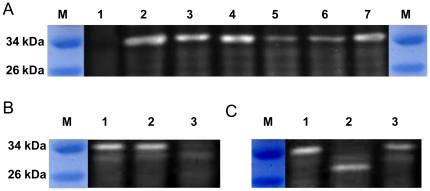
Western blot analysis of FlaA and FlaB. Whole-cell lysates were prepared from *S. oneidensis* cultures in mid-exponential growth phase, loaded equally, separated on 12% SDS-PAGE, and electrophoretically transferred to polyvinylidene difluoride (PVDF). *S. oneidensis* flagellins were probed with polyclonal antibodiesthat recognize both FlaA and FlaB and detected by chemiluminescence. In all panels, M represents the protein marker. The samples in each lane are the (A) 1, Δ*flaA*Δ*flaB* strain, 2, Δ*flaB*/*flaB* strain, 3, Δ*fliA*/*fliA* strain, 4, wild type strain, 5, Δ*fliA* strain, 6, Δ*flaB* strain, 7, Δ*flaA* strain; (B) 1, wild type strain, 2, Δ*fliD*/*fliD* strain, 3, Δ*fliD* strain; (C) 1, wild type strain, 2, Δ*pseB* strain, 3, Δ*pseB*/*pseB* strain.

The relative transcription levels of *flaA* and *flaB* agree well with the contents of their products inthe flagella filament. However, in recent studies it was shown that the relative amounts of flagellins is subject to multiple levels of regulation and modification, including post-transcriptional, posttranslational controls as well as secretion [17], [55]–[57]. To test whether mechanisms at other levels account for the contrasting phenotypes of the *flaA* and *flaB* mutants, we raised antibodies against a peptide fragment shared by FlaA and FlaB and used them to detect flagellin subunits in the Δ*flaA*, Δ*flaB*, Δ*fliA* strains and in their parental wild type strains. Western blotting revealed a single band in the wild type but not in the Δ*flaA*Δ*flaB* double mutation strain. At the same position, a band was visible in both Δ*flaA* and Δ*flaB*, indicating that the two flagellins migrated identically on sodium dodecyl sulfate-polyacrylamide gel (SDS-PAGE) (Fig. 5). This single band was at a position approximately 4∼5 kDa higher than expected for the deduced molecular masses of each of the flagellins (∼29 kDa), suggesting the possibility of posttranslational modification, mostly likely glycosylation. The band in Δ*flaA* was much stronger than that in Δ*flaB*, supporting other evidence that FlaB is produced in substantially greater amount than FlaA. Additionally, results showed that Δ*fliA* possessed the smallest number of flagellins proteins, all presumably FlaA. The immunoblotting assay also confirmed the successful complementation of mutations in *fliA* and *flaB*. It is noteworthy that the plasmid borne *flaB* promoted over-production of FlaB flagellin in comparison to the amount of this protein in the wild type.

As a considerable number of FlaA and FlaB proteins were present, a *fliD* mutant was constructed and assayed by immunoblotting to demonstrate whether these flagellin subunits are subjected to the feedback control. Results showed that the *fliD* mutant was completely non-motile on swarming or swimming plates and the plasmid borne *fliD* fully rescued the defect (Table 1, Fig. 2). Consistently, TEM studies demonstrated that the mutant cells possessed a truncated flagellum (Fig. 3), a characteristics of non-cap mutants in other bacteria [58]. Additionally, the mutation reduced the amount of flagellin drastically(lane 3 in Fig. 5B). The *fliD* gene on pHG101 complemented the *fliD* mutation resulting in similar levels of flagellin. To further investigate whether the feedback control occurs at the transcriptional level, we performed the β-Galactosidase assay to monitor the activity of the *flaA* and *flaB* promoters in the *fliD* mutant. Consistent with the Western blotting results, both promotersshowed substantially reduced activities, confirming other evidence that feedback control inhibits transcription of both genes (Fig. 4). These results indicate that both *flaA* and *flaB* are under feedback control and this control exertedprimarily at the transcription level.

### FliS interacts with FlaA and FlaB

A recent study supported a novel mechanism for flagellar gene expression is dictated by the rate of protein secretion [59]. As a consequence, intracellular levels of flagellins may have inhibitory effects on their own production. The flagellin subunits are synthesized in the cytosol and transported as monomers to the nascent end of the flagellum [1]. Before transport, chaperon FliS functions to prevent flagellin polymerization in the cytosol by binding to the C-terminal D0 domain of the flagellin protein [60]–[61]. We then asked whether FliS recognizes FlaA and FlaB differently, causing one of them to be favorably secreted.

To test this, we generated plasmid constructs capable of detecting protein-protein interactions between FliS and FlaA as well as FliS and FlaB using the BacterioMatch II Two-Hybrid System. Plasmids containing ‘bait’ (FlaA or FlaB) and ‘target’ (FliS) were co-transformed into BacterioMatch II Validation Reporter Competent Cells for interaction detection. Positive protein interactions allow growth of the reporter strain on 3-AT (see Methods). As shown in Table 3, cells containing either FlaA/FliS or FlaB/FliS were able to form colonies on 3-AT in less than 24 h, similar to cells hosting the positive control plasmid pair. These positive interactions were confirmed by growth of these colonies on plates containing both 3-AT and streptomycin (12.5 µg/ml). In contrast, cells with the negative control plasmid pair failed to produce any colonies in 40 h. These results indicate that FliS is able to interact with both FlaA and FlaB. Meanwhile, we performed the same assay on FlaG and FliS. As expected, no interactions were detected between these two proteins, providing additional evidence that FlaG is not a flagellin subunit.

**Table 3:** Bacterial two-hybrid assay of FliS-FlaA, FliS-FlaB, and SO3234-FliD.

Bait	Target	Colonies on nonselective plates^a^	Colonies on Selective plates^b^	Confirmation^c^	Result Interpretation
pBT	pTRG	201	0	–	no bait + no target – no interaction
pBT	pTRG-Gal11^P^	177	0	–	no bait + target Gal11^P^ – no interaction
pBT-LGF2	pTRG-Gal11^P^	188	159	159	bait LGF2 + target Gal11^P^ – strong interaction
pBT	pTRG-FliS	231	0	–	no bait + target PliS – no interaction
pBT-FlaA	pTRG	143	0	–	bait FlaA + no target – no interaction
pBT-FlaA	pTRG-FliS	150	47	47	Bait FlaA + target FliS – strong interaction
pBT-FlaB	pTRG	128	0	–	bait FlaB + no target – no interaction
pBT-FlaB	pTRG-FliS	192	104	104	Bait FlaB+ target FliS – strong interaction
pBT-FlaG	pTRG	222	0	–	Bait FlaG+ no target – no interaction
pBT-FlaG	pTRG-FliS	266	0	–	Bait FlaG+ target FliS – no interaction
pBT	pTRG-SO3234	163	0	–	no bait + target SO3234 – no interaction
pBT-FliD	pTRG	145	0	–	bait FliD + no target – no interaction
pBT-FliD	pTRG-SO3234	138	0	–	Bait FliD+ target SO3234 – no interaction

a M9 agar +25 µg/ml chloramphenicol +12.5 µg/ml tetracycline.

b a+5 mM 3-AT.

c b+12.5 µg/ml streptomycin.

### Both FlaA and FlaB are glycosylated

The apparent low migration rates of FlaA and FlaB on SDS-PAGE can be explained by posttranslational modification. The most abundant flagellin modification is glycosylation by the *O*-glycan pathways [17], [62]. Extensive studies on *C. jejuni* and *Helicobacter pylori* have shown that the predominant *O*-glycans on flagellins are derivatives of pseudaminic acid (Pse) or legionaminic acid (Leg) [63]. We reasoned that *S. oneidensis* uses the same strategy to modify flagellins thus promoting flagella assembly. The genome screening for flagellin glycosylation proteins identified SO3271 and SO3270 as promising candidates showing a sequence identity of greater than 30% to known components of the Pse and Leg pathways. SO3271and SO3270, predicted to be co-transcribed, share approximately 66% and 36% identity with PseB (*C. jejuni* CJ1293 and *H. pylori* HP0840) and PseC (*C. jejuni* CJ1294 and *H. pylori* HP0366), respectively. PseB (NAD(P)-dependent dehydratase/epimerase) and PseC (PLP-dependent aminotransferase), catalyze the first two steps in the pathway, converting UDP-α-D-GlcNac to UDP-2-acetamido-2,6-dideoxyb-l-*arabino*-hexos-4-ulose, then to UDP-4-amino-4,6-dideoxy-b-*L*-AltNAc [64].

To test the role of PseB in flagellin modification, we created an *SO3271* (*pseB*) in-frame deletion strain. Like Δ*pseB* of *H. pylori*, the mutant was non-motile on swimming or swarming plates (Table 1, Fig. 2), indicating the essential role of this protein in motility [65]. Complementation with *SO3271* on pHG101restored motility comparable to the parental wild type (Fig. 2, Table 1). To confirm that the motility phenotype resulted from flagellin modification, extracts from the mutant were subjected to Western blotting analysis (Fig. 5C). Flagellins from theΔ*pseB strain* migrated much faster than the comparable proteins from the wild type. The position exactly matched the calculated molecular mass for both FlaA and FlaB, illustrating that flagellins in Δ*pseB*are in un-modified form. Additionally, glycosylated flagellins were not detected in the mutant strain in the analysis, suggesting that glycosylation by the PseBpathwayfunctions for both FlaA and FlaB. On the basis of similarities in sequence and functionality between SO3271 and PseB, we designated *SO3271* of *S. oneidensis* as *pseB*.

### SO3234 is importantfor motility but may not be the chaperon of FliD

In *E. coli* and *S. typhimurium*, operon *fliDST* encodes the cap FliD, the flagellin chaperon FliS, and the FliD chaperon FliT [1]. Since FliT is a regulator that inhibits the flagellar master regulatory proteins FlhDC by direct binding [66], FliD can function as an anti-regulator by sequestering FliT from FlhDC [1]. Unlike the *fliD* defect mutantsthat possess truncated flagella and lose motility fully, the *fliT* mutants produce flagellar structures indistinguishable from those produced by their parental wild type [67]. Consequently, the mutants are as motile as the wild type [68]. In *S. oneidensis*, the genes downstream of *fliD* are *SO3234* and *fliS*. Intriguingly, although this type of organization is also observed in a variety of bacteria including *V. cholera*, little has been done to elucidate the role of SO3234 and its analogs in flagellar assembly. The question arose whether SO3234 (106 a.a.)is functionally equivalent to FliT.

Our mutational analysis has validated that the annotated *fliD* indeed encodes the cap of the flagellar filament so we constructedan *S. oneidensis* strain devoid of *SO3234*. The mutant Δ*SO3234* displayed a decrease in motility to approximately 56% relative to its parental strain (Table 1, Fig. 2), in contrast to the findings that *fliT* mutants produced functional flagella in general [38], [67]–[69]. When pHG101 containing *fliD*-*SO3234*fused to the *arcA*promoter was inserted, both mutant strains had their motility fully restored (Fig. 2, Table 1). Surprisingly, the mutants appeared to produce full-length flagella (Fig. 3), indicating that the reduced motility may not be due to impaired filament formation.

To further dissect the role of SO3234, the bacterial two-hybrid system was employed to examine the direct interaction of SO3234 and FliD. The *SO3234* gene was cloned into the bait plasmid while *fliD* was used as the target, and these were co-transformed into the *E. coli* reporter strain. In contrast to the positive control, no colonies were found on plates containing 3-AT after 24 hours of incubation (Table 3). Additional incubation for 16 hours which allows the growth of cells containing weak interactors did not help. These results rule out the possibility that FliD interacts with SO3234 *in vivo*, thus confirming that SO3234 is unlikely to be the chaperone for FliD.

## Discussion

Flagellar synthesis in *S. oneidensis*has been presumed to be similar to that in *V. cholera*, the research paradigm for polarly flagellated bacteria, despite apparent permutations in the flagellar gene contents and organization betweenthe two genomes [13], [33], [34], [70]. As a result, only a couple of studies have been done in deciphering the *S. oneidensis*motility system in contrast to extensive investigation on its anaerobic respiration and metal reduction for more than two decades [21], [26], [36], [39]. In this study, we have performed a relatively comprehensive genetic analysis of the polar flagellumin *S. oneidensis*, generating three contributions to the current understanding of polar flagellar synthesis. First, we provide insights about the two flagellin subunits in respect to their functionality and regulation. Second, we demonstrated that both FlaA and FlaB are glycosylated by anovel pathway utilizing PseB. Third, we present data that SO3234 may not be the chaperon for FliT, arguing that the polar flagella may not require chaperons for their assembly.

Most of polar flagellated bacteria possess multiple flagellins which are similar to one another [11], [49], [71]–[73]. These microbes can be readily classified into two groups based on whether functionally predominant flagellins are present. *V. parahaemolyticus*, *Caulobacter crescentus*, and *Aeromonas hydrophila*are representatives of the group without dominant filament subunits [11], [71], [73]. For bacteria in the other group, the removal of the predominant flagellin subunits causes a loss in motility [11], [49], [74]–[75]. Meanwhile, the minor flagellins are so insignificant that their mRNAs (*C. jejuni*) may not be above a detectable level [49], [74]. Consistently, major flagellins outnumber the minor ones hundreds or thousands of times. This phenomenon has largely been accredited to the fact that flagellin genes are under the control of different promoters, recognized by specialized regulatory proteins and/or sigma factors. In *V. cholera*, the major flagellin gene is σ^54^–dependent [11] while *C. jejuni* and *C. coli* employs σ^28^ to direct expression of the predominant flagellin [49], [74]. In *S. oneidensis*, disruption of either flagellin gene fails to lead to a complete non-motile phenotype although σ^28^–dependent FlaB is evidently more important than FlaA in motility. More intriguingly, transcription of both genesis considerable as is the amounts of FlaA and FlaB. Collectively, compared to the other studied polarly flagellated bacteria *S. oneidensis* seems to assemble flagella with ‘weaker’ major and ‘stronger’ minor flagellins, thus representing a novel model.

Flagella production is a metabolically costly endeavor for bacterial cells and therefore is tightly regulated, particularly at the transcriptional level. To prevent production of unnecessary flagellar subunits, additionalfeedback loops are exploited to ensure that during flagellar assembly every stage is signaled prior to synthesis of the components for the next stage [76]. In a recent study, it was reported that secretion efficiency of flagellins plays an important role [44]. The secretion signal modulating efficiency is located in the amino-terminal sequence, more precisely the N-terminal D_0_ domain of flagellin [77]. For example, protein-specific conserved residues are identified between *C. jejuni* FlaA and FlaB in the D_0_ domain by sequence alignment of both proteins from multiple strains [44]. These protein-specific residues dictate the secretion efficiency. In *S. oneidensis*, however, this may not be the case. Amino acid sequence alignment of the D_0_ domains of FlaA and FlaB from 23 sequenced *Shewanella* strains reveals a similar degree of conservation in N- and C-terminal D_0_ domains. More importantly, no protein-specific residues are found within either domain, suggesting that secretion of FlaA and FlaB may be similar if it is dependent on this terminal sequence. In addition, the chaperon FliS may not make a difference because the D0 domain binding regionsof these two flagellinsshare the highest identity [60]–[61]. However, there are some features residing in these two flagellins that distinguish them because the greater motility of the *S. oneidensis* strain with the over-expressed FlaB is only found in the presence of FlaA. This certainly merits further investigation.

Although great efforts have been made to increase our understanding of the polar flagellar system in recent years,the function of FliT is still elusive. In bacteria with lateral flagella such as *Salmonella*, chaperon FliT ofFliD inhibits the transcriptional activator FlhD_4_C_2_, suggesting that FliT is a regulatory component conserved throughoutthese flagellar systems [66], [78]–[79]. Interestingly, despite multiple roles of FliT the mutation in this gene did not affect either flagellar structure or motility [67] but export of FliD was significantly reduced in the mutant [68].

Unlike most bacteria with multiple separating segments for flagellar genes, *S. oneidensis* allocates only one major region, making it an ideal organism for identification of unknown flagellar genes. Among a few unknown flagellar genes, *SO3234* appears most likely to be the counterpart of FliT because it shares conserved synteny with *fliT* in peritrichously flagellated bacteria and is also similar in size. We found that although the *SO3234* null mutant showed reduced motility, it produced a flagellum indistinguishable from that in its parental strain. This observation is resonant with the finding by Capdevila *et al.* [38], indicating that the *SO3234*mutant is phenotypically different from *fliT* mutants. Furthermore, SO3234 is unable to interact with FliD, eliminating the possibility of chaperoning FliD. Given that most of the polarly flagellated bacteria also lack the second interaction partner of FliT, FlhC, we therefore assume that FliT is unlikely to be present in this group of microorganism. However, whether FliT is only conserved among enteric bacteria as suggested by Imada *et al*. [79] demands further investigation because FlhDC has been identified in a polarly flagellated bacterium, *Burkholderia glumae* [25].

**Figure 6:**
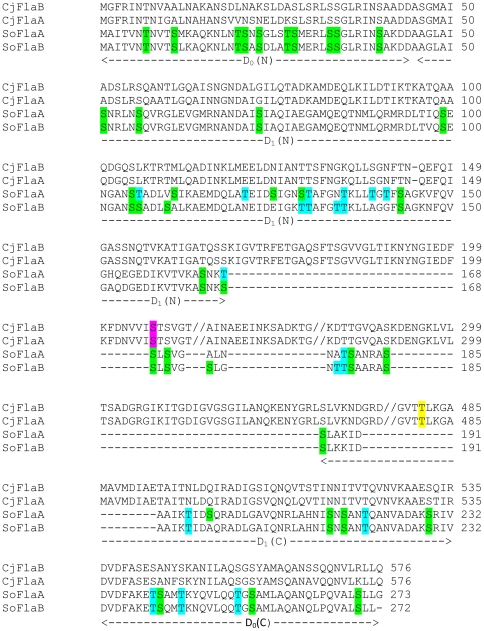
Potential glycosylation sites in FlaA and FlaB of *S. oneidensis*. *S. oneidensis* flagellins (SoFlaA and SoFlaB) are aligned with FlaA and FlaB of *C. jejuni* 81–176 (CjFlaA and CjFlaB). The domain information is based on the domains of *Salmonella enterica* serovar Typhimurium flagellin. Positions of the glycosylation sites of CjFlaA are in purple (S206, found in *S. oneidensis*) and in yellow (T481, missing in *S. oneidenisis*, only one is shown). Serine and threonineresidues in *S. oneidensis* flagellins are in green and blue, respectively.

Strikingly, both flagellins of *S. oneidensis* lack the D_2_–D_3_ domains, retaining only one glycosylation siteas identified in the flagellins of *C. jejuni* 81–176 (Fig. 6). Assuming that the basic structure of *S. oneidensis* flagellin is similar to that of *Salmonella* [20], it is unlikely that the majority of glycosylation sites are located in the D_0_–D_1_ domains because they are not exposed. If this holds, there are at most 7 residues suitable for glycosylation. Consequently, it is almost certain that Pse (MW = 316) is not the pathway glycosylating flagellins because it needs at least 15 sites to account for the observed mass increase of 4∼5 kDa. However, *S. oneidensis* apparently employs PseB to initiate glycosylation. This is not surprising because the bacterium has a complete set of enzymes and transporters for GlcNac metabolism to generate abundant substrates [80]. Therefore, flagellar glycosylation in *S. oneidensis* may be carried out by anovel pathway by components which may evolve independently as promiscuous enzymes that work in multiple pathways. Additional work is in progress to identify other genes in the pathway and to dissect the role of glycosylation in the flagellar assembly in *S. oneidensis*.

## Methods

### Bacterial strains, plasmids, and culture conditions

A list of all bacterial strains and plasmids used in this studyis given in Table 4 [81]. For genetic manipulation, *E. coli* and *S. oneidensis* strains weregrown under aerobic conditions in Luria-Bertani (LB) medium at 37 and 30°C,respectively. When needed, the growth medium was supplementedwith antibiotics at the following concentrations: ampicillin at50 µg/ml, kanamycin at50 µg/ml,and gentamycin at 15 µg/ml.

**Table 4:** Strains and plasmids used in this study.

Strain or plasmid	Description	Reference or source
*E. coli* strain		
WM3064	Donor strain for conjugation; *ΔdapA*	[81]
*S. oneidensis* strains		
MR-1	Wild-type	ATCC 700550
HG3210	*fliA* deletion mutant derived from MR-1; *ΔfliA*	This study
HG3234	*SO3234* deletion mutant derived from MR-1; *ΔSO3234*	This study
HG3235	*fliD* deletion mutant derived from MR-1; *ΔfliD*	This study
HG3237	*flaB* deletion mutant derived from MR-1; *ΔflaB*	This study
HG3238	*flaA* deletion mutant derived from MR-1; *ΔflaA*	This study
HG3240	*flgK* deletion mutant derived from MR-1; *ΔflgK*	This study
HG3237-8	*flaB* and *flaA* double deletion mutant derived from MR-1; *ΔflaBΔflaA*	This study
HG3210-3238	*fliA* and *flaA* double deletion mutant derived from MR-1; *ΔfliAΔflaA*	This study
Plasmids		
pDS3.0	Ap^r^, Gm^r^, derivative from suicide vector pCVD442	[82]
pDS-FLIA	pDS3.0 containing the PCR fragment for deleting *fliA*	This study
pDS-3234	pDS3.0 containing the PCR fragment for deleting *3234*	This study
pDS-FLID	pDS3.0 containing the PCR fragment for deleting *fliD*	This study
pDS-FLAA	pDS3.0 containing the PCR fragment for deleting *flaA*	This study
pDS-FLAB	pDS3.0 containing the PCR fragment for deleting *flaB*	This study
pDS-FLGK	pDS3.0 containing the PCR fragment for deleting *flgK*	This study
pTP327	Broad host *lacZ* reporter vector	[43]
pTP327-FLAAp	pTP327 containing the *S. oneidensisflaA* promoter	This study
pTP327-FLABp	pTP327 containing the *S. oneidensisflaB* promoter	This study
pBBR1MCS-2	Broad host Km^r^ vector used for complementation	[83]
pHG101	Promoterless broad host Km^r^ vector used for complementation	This study
pHG102	pHG101 containing the *arcA* promoter	This study

### Construction and complementation of in-frame deletion mutants

In this study, in-frame deletion strains were constructed using the Fusion PCR method [82]. Primers used for generating PCR products for mutagenesis are available upon request. In brief, two fragments flanking the targeted gene were amplifiedindependently first and joined together by the second round of PCR. The resulting fusion fragment for each individual gene was introduced into plasmidpDS3.0. The resulting mutagenesis vector was transformed into *E. coli* WM3064, and then transferred into *S. oneidensis* by conjugation. Integration of the mutagenesis constructinto the chromosome was selected by gentamycin resistanceand confirmed by PCR. Verified transconjugants were grown in LB broth in the absence of NaCl and plated on LB supplemented with 10% of sucrose. Gentamycin-sensitive and sucrose-resistant colonies were screened by PCR for deletion of the targeted gene. The deletion mutation was then verified by sequencing of the mutatedregion.

To facilitate complementation experiments, two plasmids were constructed in this study. The first plasmid, pHG101, was formed by replacing *lacZα* and its promoter region on pBBR1MCS with the amplified MCS of the same plasmid [83]. The second plasmid, pHG102, was derived from pGH101 by placing the *S. oneidensis arcA* promoter in the front of the MCS. For complementation of genes next to their promoter, a fragment containing the targeted gene and its native promoter was generated by PCR and cloned into pHG101. For other genes, the targeted gene was amplified and inserted into MCS of pHG102 under the control of the *arcA* promoter. Introduction of each verified complementation vector into the corresponding mutant wasdone by mating with WM3064 containing the vector, andconfirmed by plasmid extraction and restriction enzyme mapping.

### Physiological characterization

Growth of deletion strains in LB was measured by recording cell densities of cultures at 600 nm under aerobic conditions in triplicate with strain MR-1 as the control. To determine the swarm and swim motility of mutants, one microliter of an overnight culture was spotted in the middleof a swarm LB plate (0.5% agar) or a swim LB plate (0.25%agar) and allowed to dry for 1 h at room temperature. All plateswere incubated at 30°C for 10 h or as noted otherwise. Forphase-contrast microscopic analysis, swarmor swim cells werescraped from the leading edges of each swarm and then visualized inNB or saline on a glass slide.

### Electron microscopy visualization

For transmission electron microscopy (TEM), cells grown overnighton 1% tryptone agar plates were suspended in sterile distilledwater, spread onto carbon-Formvar copper grids, and then negativelystained with 1% phosphotungstic acid (pH 7.4). Preparations were viewedunder a CM12 Philips TEM.

### β-Galactosidase activity assay

A *lacZ* reporter system for *S. oneidensis* has been developed [43]. To construct the *flaA-lacZ* and *flaB-lacZ* reporters, the *flaA* and *flaB* promoter DNA fragments were generated by PCR (primers available upon request), cloned into pTP327, and verified by sequencing. The reporter plasmids were moved into *S. oneidensis*Δ*fliA* or the MR-1 strain by conjugation. Cells in the log phase (30°C, OD_600_ = 0.4) were harvested by centrifugation, washed with PBS(phosphate buffered saline), and treated with lysis buffer (0.25 M Tris/HCl, (pH 7.5), 0.5% Trion-X100). The resulting soluble protein was collected after centrifugation and used for enzyme assaysemploying the High Sensitivity β-Galactosidase Assay Kit (Stratagene) according to manufacturer's instructions. β-galactosidaseactivity was determined by monitoring color development at 575 nm every minute for 30 min by using a Synergy 2 Multi-Detection Microplate Reader. The protein concentration of the cell lysates was determined using a Bradford assay with BSA as a standard (Bio-Rad).

### Bacterial two-hybrid assay

The BacterioMatch II Two-Hybrid system (Stratagene) wasused to investigate protein-protein interaction *in vivo* in *E. coli* cells according to manufacturer's instructions. Briefly, plasmid constructs were created by cloning the target and bait proteins in the pTRG and pBT vectors and verified by sequencing. The resultant plasmids were used to co-transform BacterioMatch II Validation Reporter Competent Cells on M9 salt agar plates containing 25 µg/ml chloramphenicoland 12.5 µg/ml tetracycline with or without 3-amino-1,2,4-triazole (3-AT). pBT-LGF2, pTRG-Gal11P,and empty pBT and pTRG constructs were used as positive andnegative controls. The plates were incubated at 37°C for 24 h and then moved to room temperature for an additional 16 h (the colonies indicating a positiveinteraction usually appeared between 18 and 24 h). The positiveinteractions were confirmed by streaking colonies on platescontaining both 3-AT and streptomycin (12.5 µg/ml).

### Immunoblotting assay

Rabbit polyclonal antibodies against a fragment of FlaB (CRDLTIQSENGANST) were prepared in accordance with standard protocols provided by the manufacturer (Genscript) and used for immunoblotting analysis. Bacterial cells in log phase (30°C, OD_600_ = 0.4) were used. For these experiments, cell samples were washed once with TE buffer (10 mM Tris [pH 8.0], 1 mM EDTA), and resuspended to an optical density at 600 nm (OD_600_) of 1.0 inlysis buffer (50 mM Tris [pH 8.0], 1 mM EDTA, 100 mM NaCl). The total protein concentration of the cell lysateswas then determined by the bicinchoninic acid assay (PierceChemical). Samples were loaded onto SDS-12% polyacrylamide gels and either stained with Coomassie brilliant blue or electrophoretically transferred to polyvinylidene difluoride (PVDF) according to the manufacturer's instructions (Bio-Rad). The gels were blotted for 1 h at 50 V using a Criterion blotter(Bio-Rad). The blotting membrane was probed with anti-FlaB antibody, followed by a 1∶10,000 dilution of goat anti-mouseimmunoglobulin G-alkaline phosphatase conjugate, and thealkaline phosphatase was detected using a chemiluminescence Western blotting kit (Roche Diagnostics) inaccordance with the manufacturer's instructions. Images were visualized with the UVP Imaging System.

### Identification of flagellar genes under the control of σ^28^

The operons under the control of σ^28^ in *E. coli*, *Salmonella typhimurium* and *V. cholerae* were from references [13], [53]. Common σ^28^ binding motif identification, weight matrix construction, and genome screening were performed as described previously [43].

## Supporting Information

Figure S1 **Organization of flagellin genes in** ***Shewanellae*** **.** Among sequenced *Shewanella* strains,12 including *S. oneidensis* possess two genes encoding flagellins of 265–275 a.a.. The second largest group consists of 5 strains including *Shewanella* sp. MR-4, MR-7, *S. benthica*, *S. violacea*, and *S. frigidimarina*, whosegenes encode flagellin subunits of 465–482 a.a.. *S. baltica* OS185 and OS195 possess four genes encoding flagellins, which appear to have resulted from transposition events. The other two strains *S. pealeana* and *S. piezotolerans* contain genes encoding flagellins of 393–394 a.a. and 434–463 a.a., respectively.
(PDF)

Table S1 **σ^28^-dependent genes in** ***Shewanella oneidensis.***
(PDF)
