# Supplementary material for: Genetic and Molecular Characterization of Flagellar Assembly in Shewanella oneidensis
Source: PLoS One. 2011 Jun 22;6(6):e21479. doi: 10.1371/journal.pone.0021479 (PMC3120886; doi:10.1371/journal.pone.0021479)
Supplement: Figure S1 — Organization of flagellin genes in Shewanellae . Among sequenced Shewanella strains,12 including S. oneidensis possess two genes encoding flagellins of 265–275 a.a.. The second largest group consists of 5 strains including Shewanella sp. MR-4, MR-7, S. benthica, S. violacea, and S. frigidimarina, whosegenes encode flagellin subunits of 465–482 a.a.. S. baltica OS185 and OS195 possess four genes encoding flagellins, which appear to have resulted from transposition events. The other two strains S. pealeana and S. piezotolerans contain genes encoding flagellins of 393–394 a.a. and 434–463 a.a., respectively. (PDF) [file pone.0021479.s001.pdf]

Species	Organization of flagellin genes	Average Size of flagellins
S. oneidensis	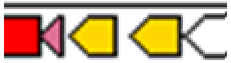	~270 a.a
S. baltica OS185	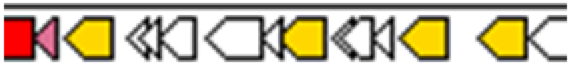	~270 a.a
S. baltica OS195	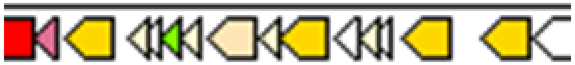	~270 a.a
S. piezotolerans	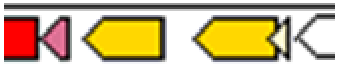	~434 and 463 a.a
S. violacea	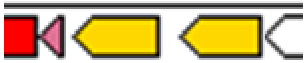	~465 a.a
S. benthica	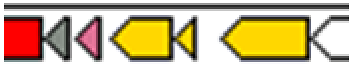	~344 a.a
S. pealeana	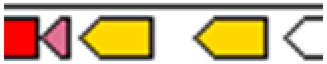	~393 a.a
