# Supplementary material for: Genetic and Molecular Characterization of Flagellar Assembly in Shewanella oneidensis
Source: PLoS One. 2011 Jun 22;6(6):e21479. doi: 10.1371/journal.pone.0021479 (PMC3120886; doi:10.1371/journal.pone.0021479)
Supplement: Table S1 — σ28-dependent genes in Shewanella oneidensis. (PDF) [file pone.0021479.s002.pdf]

Table S1 σ^{28}-dependent genes in *Shewanella oneidensis*

1	**seq_id**	**ft_name**	**strand**	**start**	**end**	**sequence**	**weight**	**Pval**	**ln_Pval**
2	rnt	TAAAGkwtkyrhwhhdcytGCCGATAa	R	85	111	TAAAGGAACTAAAAAGTTTGCCGATAA	17.7	1.00E-09	-20.706
3	motY	TAAAGkwtkyrhwhhdcytGCCGATAa	D	83	109	TAAAGGAACTAAAAAGTTTGCCGATAA	17.7	1.00E-09	-20.706
4	SO_3257	TAAAGkwtkyrhwhhdcytGCCGATAa	D	135	161	TAAAGTTATTGGCACAGCTGTCGATAA	13.6	1.50E-07	-15.727
5	SO_3258	TAAAGkwtkyrhwhhdcytGCCGATAa	R	113	139	TAAAGTTATTGGCACAGCTGTCGATAA	13.6	1.50E-07	-15.727
6	SO_3261	TAAAGkwtkyrhwhhdcytGCCGATAa	D	11	37	TAAAGCTTCCCTGTCATTTGCCGTTAA	13.2	2.20E-07	-15.338
7	flaB	TAAAGkwtkyrhwhhdcytGCCGATAa	D	148	174	TAAAGATAAAAAAGCTGCTGCCGCTAT	12.6	3.80E-07	-14.775
8	SO_3151	TAAAGkwtkyrhwhhdcytGCCGATAa	D	3	29	TAAAGTGTGTATACTGCGCGCCTTTAA	12.3	5.00E-07	-14.503
9	SO_0740	TAAAGkwtkyrhwhhdcytGCCGATAa	D	165	191	TAAAGTTGCGGATAATATTGCCGAAAA	11.1	1.40E-06	-13.466
10	SO_2500	TAAAGkwtkyrhwhhdcytGCCGATAa	D	341	367	TAAAGTTCTTGTTCAACTGGTCGATTA	11.1	1.40E-06	-13.466
11	SO_4229	TAAAGkwtkyrhwhhdcytGCCGATAa	D	342	368	TAAAAATTAGGCAACTCCGGCCGATAA	11.1	1.40E-06	-13.466
12	ispA	TAAAGkwtkyrhwhhdcytGCCGATAa	R	2	28	TAAACTTGCTGAGTGCTTGTCCGATAA	10.6	2.10E-06	-13.056
13	pomA	TAAAGkwtkyrhwhhdcytGCCGATAa	D	217	243	TAAACTTGCTGAGTGCTTGTCCGATAA	10.6	2.10E-06	-13.056
14	hsdM-2	TAAAGkwtkyrhwhhdcytGCCGATAa	D	25	51	TAAACGATAGGCCATGACAGCCAATAA	10.1	3.20E-06	-12.658
15	flgM	TAAAGkwtkyrhwhhdcytGCCGATAa	D	8	34	TAAAGCTTTTTGATGAGCTGCCGATAC	9.9	3.70E-06	-12.502
16	nhaD	TAAAGkwtkyrhwhhdcytGCCGATAa	R	45	71	TCAAGTTAGTCTAAAAATAGCCAATAA	9.6	4.70E-06	-12.271
17	rne	TAAAGkwtkyrhwhhdcytGCCGATAa	D	92	118	TAAAGTTTTTCTCACCGATTTCGAAAT	9.5	5.10E-06	-12.194
18	SO_2918	TAAAGkwtkyrhwhhdcytGCCGATAa	R	57	83	TAAACGGGGCGAAGAGGAAGTCGATAT	9.3	5.90E-06	-12.043
19	SO_3895	TAAAGkwtkyrhwhhdcytGCCGATAa	R	277	303	TAAACGATTTAAGCCGAAAACCGAAAT	9.1	6.80E-06	-11.894
20	rtxB	TAAAGkwtkyrhwhhdcytGCCGATAa	R	44	70	ACAAGTATCCACCACGATGGCCGATAT	8.9	7.90E-06	-11.746
21	SO_0819	TAAAGkwtkyrhwhhdcytGCCGATAa	R	38	64	TAAAGTATCCCTGTTTTTTTACGCTAT	8.9	7.90E-06	-11.746
22	SO_4512	TAAAGkwtkyrhwhhdcytGCCGATAa	R	255	281	TAATTAAATCATCTTGCTTGCCGTTAA	8.7	9.20E-06	-11.6
23	SO_3944	TAAAGkwtkyrhwhhdcytGCCGATAa	R	120	146	TAAATGTCGCGTTCACATGGTCAATAA	8.3	1.20E-05	-11.313
24	SO_1136	TAAAGkwtkyrhwhhdcytGCCGATAa	R	348	374	GAAAGTTTACGCAGAGTTGGCAGTTAA	8.2	1.30E-05	-11.242
25	gcvP	TAAAGkwtkyrhwhhdcytGCCGATAa	R	94	120	TAAACCTTGTTCCAGATTTGCCGTAAC	8.1	1.40E-05	-11.171
26	SO_2083	TAAAGkwtkyrhwhhdcytGCCGATAa	D	339	365	TAAAGTCTATGGCAAATCGACCGATAT	8.1	1.40E-05	-11.171

27	SO_0808	TAAAGkwtkyrhwhhdcytGCCGATAa	D	84	110	TAAAGGTCATATCGCACTCGACGATCT	7.9	1.60E-05	-11.032
28	SO_0891	TAAAGkwtkyrhwhhdcytGCCGATAa	D	54	80	TAAACGTCCTTCAAAATAAATCGCTAA	7.8	1.70E-05	-10.962
29	SO_2314	TAAAGkwtkyrhwhhdcytGCCGATAa	D	267	293	TAAACGTCCTTCAAAATAAATCGCTAA	7.8	1.70E-05	-10.962
30	SO_3501	TAAAGkwtkyrhwhhdcytGCCGATAa	D	195	221	TAAACGTACCAATCCCCGTACCGATGG	7.8	1.70E-05	-10.962
31	SO_A0100	TAAAGkwtkyrhwhhdcytGCCGATAa	R	118	144	TAAACGTCCTTCAAAATAAATCGCTAA	7.8	1.70E-05	-10.962
32	aroA	TAAAGkwtkyrhwhhdcytGCCGATAa	D	98	124	TAATCTGGCAAAAAAGCCTGCCGAAAA	7.7	1.90E-05	-10.893
33	asmA	TAAAGkwtkyrhwhhdcytGCCGATAa	D	4	30	TAAAAAAATTCAATATGTTATCGATAA	7.7	1.90E-05	-10.893
34	aspC-2	TAAAGkwtkyrhwhhdcytGCCGATAa	R	243	269	TAATCTGGCAAAAAAGCCTGCCGAAAA	7.7	1.90E-05	-10.893
35	SO_4134	TAAAGkwtkyrhwhhdcytGCCGATAa	R	46	72	TCAAGTTAACCTTCACCGCTTCGATAC	7.6	2.00E-05	-10.824
36	udp	TAAAGkwtkyrhwhhdcytGCCGATAa	D	317	343	TCAAGTTAACCTTCACCGCTTCGATAC	7.6	2.00E-05	-10.824
37	flgB	TAAAGkwtkyrhwhhdcytGCCGATAa	R	90	116	TGAACAAAGCAATTCATGTGCCAATAC	7.4	2.30E-05	-10.688
38	SO_0501	TAAAGkwtkyrhwhhdcytGCCGATAa	R	134	160	TAAATGAGGTATATTCCATAGCGCTAA	7.4	2.30E-05	-10.688
39	SO_0712	TAAAGkwtkyrhwhhdcytGCCGATAa	D	209	235	TAAATTCGTTGATTAAAATGGCGATAA	7.4	2.30E-05	-10.688
40	SO_3418	TAAAGkwtkyrhwhhdcytGCCGATAa	R	75	101	TAAACGTCCCTATTTACCTGCTGATTA	7.4	2.30E-05	-10.688
41	SO_4470	TAAAGkwtkyrhwhhdcytGCCGATAa	R	148	174	TAAGGTGTGACCGAATGATGGCGTTAA	7.4	2.30E-05	-10.688
42	radA	TAAAGkwtkyrhwhhdcytGCCGATAa	R	104	130	TTAAGTAACTGTGAATACTGCCGACAT	7.3	2.40E-05	-10.62
43	SO_1225	TAAAGkwtkyrhwhhdcytGCCGATAa	D	29	55	TTAAGTAACTGTGAATACTGCCGACAT	7.3	2.40E-05	-10.62
44	SO_1582	TAAAGkwtkyrhwhhdcytGCCGATAa	D	106	132	TATACAAATCGTTAAATTAGGCGATAA	7.3	2.40E-05	-10.62
45	fadB	TAAAGkwtkyrhwhhdcytGCCGATAa	D	105	131	TAAATTTATTCAATTTACCGCCAATTA	7.2	2.60E-05	-10.553
46	pemI	TAAAGkwtkyrhwhhdcytGCCGATAa	R	136	162	TAAAGCCTCCTATTCACTCGCCATTAA	7.2	2.60E-05	-10.553
47	pepQ	TAAAGkwtkyrhwhhdcytGCCGATAa	R	245	271	TAAATTTATTCAATTTACCGCCAATTA	7.2	2.60E-05	-10.553
48	SO_2768	TAAAGkwtkyrhwhhdcytGCCGATAa	R	36	62	AAAAGTTTGTAAGCTAAGTGTCGGTAT	7.2	2.60E-05	-10.553
49	SO_2769	TAAAGkwtkyrhwhhdcytGCCGATAa	D	166	192	AAAAGTTTGTAAGCTAAGTGTCGGTAT	7.2	2.60E-05	-10.553
50	SO_3688	TAAAGkwtkyrhwhhdcytGCCGATAa	D	30	56	TAAACGTGGCAATTAGGTGGAAGTTAA	7.2	2.60E-05	-10.553
51	ftn	TAAAGkwtkyrhwhhdcytGCCGATAa	R	11	37	TATATTTACCCAACCCTTTGCCCATAA	7.1	2.80E-05	-10.486
52	moeA	TAAAGkwtkyrhwhhdcytGCCGATAa	D	268	294	TATATTTACCCAACCCTTTGCCCATAA	7.1	2.80E-05	-10.486
53	SO_0134	TAAAGkwtkyrhwhhdcytGCCGATAa	D	65	91	TAAACCCGCCATAAAAACTGCCGCTAA	7.1	2.80E-05	-10.486

54	SO_1158	TAAAGkwtkyrhwhhdcytGCCGATAa	D	37	63	TAAAGGATACCAGCTCTAGGCTATTAA	7.1	2.80E-05	-10.486
55	SO_1159	TAAAGkwtkyrhwhhdcytGCCGATAa	R	156	182	TAAAGGATACCAGCTCTAGGCTATTAA	7.1	2.80E-05	-10.486
56	SO_2714	TAAAGkwtkyrhwhhdcytGCCGATAa	R	47	73	TAAAATAACCGCACCGTGAAACGATAT	7.1	2.80E-05	-10.486
57	SO_0910	TAAAGkwtkyrhwhhdcytGCCGATAa	R	266	292	TAAAGAATGAGAAAGCTAAAACGATAT	7	3.00E-05	-10.419
58	SO_0341	TAAAGkwtkyrhwhhdcytGCCGATAa	D	110	136	TAAACTTGCTTTGGTTACAGCTGCTAA	6.9	3.20E-05	-10.353
59	SO_0062	TAAAGkwtkyrhwhhdcytGCCGATAa	R	194	220	TCAAGTTTTCATCTGCTTTAGCGATAT	6.8	3.40E-05	-10.287
60	SO_0641	TAAAGkwtkyrhwhhdcytGCCGATAa	R	111	137	TAAAGGCTTCAATCCGAGAACCGTTCA	6.8	3.40E-05	-10.287
61	SO_0643	TAAAGkwtkyrhwhhdcytGCCGATAa	D	258	284	TAAAGGCTTCAATCCGAGAACCGTTCA	6.8	3.40E-05	-10.287
62	SO_0672	TAAAGkwtkyrhwhhdcytGCCGATAa	R	7	33	TAAAGTAATCTTTGATCGTGTAAATAA	6.8	3.40E-05	-10.287
63	SO_1980	TAAAGkwtkyrhwhhdcytGCCGATAa	D	297	323	TCATGTAGACAACAAGGGCGCCGTTAA	6.8	3.40E-05	-10.287
64	SO_2263	TAAAGkwtkyrhwhhdcytGCCGATAa	D	131	157	TCAAGATAGTGCGTTAGATCCCGATAT	6.8	3.40E-05	-10.287
65	SO_2439.2	TAAAGkwtkyrhwhhdcytGCCGATAa	D	19	45	TAAAAAAACCGTTATAACGGCTGATAA	6.8	3.40E-05	-10.287
66	SO_3099	TAAAGkwtkyrhwhhdcytGCCGATAa	D	156	182	TAACCTTAATTAAAACCTTGCCTTTAA	6.8	3.40E-05	-10.287
67	SO_3328	TAAAGkwtkyrhwhhdcytGCCGATAa	D	32	58	TGACGTTACAGCAATATATGCCGTTAA	6.8	3.40E-05	-10.287
68	SO_3331	TAAAGkwtkyrhwhhdcytGCCGATAa	R	213	239	TGACGTTACAGCAATATATGCCGTTAA	6.8	3.40E-05	-10.287
69	ppx	TAAAGkwtkyrhwhhdcytGCCGATAa	D	211	237	TAAAGTGCGTTCTCAAATTGCCATTTA	6.7	3.60E-05	-10.222
70	SO_0066	TAAAGkwtkyrhwhhdcytGCCGATAa	D	84	110	TAAACTGACTGACAAATTTGAGGCTAA	6.7	3.60E-05	-10.222
71	SO_0670	TAAAGkwtkyrhwhhdcytGCCGATAa	R	31	57	TAAACTGGGTTATCTTGGATGCGTTAA	6.7	3.60E-05	-10.222
72	SO_0671	TAAAGkwtkyrhwhhdcytGCCGATAa	D	159	185	TAAACTGGGTTATCTTGGATGCGTTAA	6.7	3.60E-05	-10.222
73	SO_0925	TAAAGkwtkyrhwhhdcytGCCGATAa	D	44	70	CAAACTTACTCTATTGTAAGCCATTAA	6.7	3.60E-05	-10.222
74	SO_0926	TAAAGkwtkyrhwhhdcytGCCGATAa	R	12	38	CAAACTTACTCTATTGTAAGCCATTAA	6.7	3.60E-05	-10.222
75	SO_1445	TAAAGkwtkyrhwhhdcytGCCGATAa	D	50	76	TAAACGATAGCGGCATGTCGCCGCTAT	6.7	3.60E-05	-10.222
76	SO_1710	TAAAGkwtkyrhwhhdcytGCCGATAa	D	128	154	TAAACTCTCGCCCACTTTGGCCGACAA	6.7	3.60E-05	-10.222
77	SO_1766	TAAAGkwtkyrhwhhdcytGCCGATAa	D	281	307	TAAATTAATAGTACAGGTTGAAGATAA	6.7	3.60E-05	-10.222
78	SO_2409	TAAAGkwtkyrhwhhdcytGCCGATAa	R	203	229	TAAACGTTTAATTATGATAAACAATAA	6.7	3.60E-05	-10.222
79	SO_1056	TAAAGkwtkyrhwhhdcytGCCGATAa	D	35	61	TCAAGGTTGAATTGCACCCGCCAATAG	6.6	3.90E-05	-10.156
80	SO_1100	TAAAGkwtkyrhwhhdcytGCCGATAa	D	162	188	AAAAGAGTGCGAATTTCATGCGGTTAA	6.6	3.90E-05	-10.156

81	SO_4038	TAAAGkwtkyrhwhhdcytGCCGATAa	R	127	153	TTATGGTGTTATTACCGCAGCCGTTAA	6.6	3.90E-05	-10.156
82	SO_1948	TAAAGkwtkyrhwhhdcytGCCGATAa	R	174	200	TATACGTTCTAATATTGGCGACGACAA	6.5	4.10E-05	-10.091
83	SO_1949	TAAAGkwtkyrhwhhdcytGCCGATAa	D	21	47	TATACGTTCTAATATTGGCGACGACAA	6.5	4.10E-05	-10.091
84	SO_3301	TAAAGkwtkyrhwhhdcytGCCGATAa	R	248	274	TAAGCAATGTTTAAAATTGGCCAATAT	6.5	4.10E-05	-10.091
85	SO_4727	TAAAGkwtkyrhwhhdcytGCCGATAa	R	36	62	TAAAGTTAACCATCCTTGTTACTTTAA	6.5	4.10E-05	-10.091
86	SO_A0042	TAAAGkwtkyrhwhhdcytGCCGATAa	R	26	52	TAAAGTAATTCACCACTTTTTTGATAT	6.5	4.10E-05	-10.091
87	SO_1728	TAAAGkwtkyrhwhhdcytGCCGATAa	R	312	338	TAAACAAATAAATCTGGCCACCGATTT	6.4	4.40E-05	-10.027
88	SO_3877	TAAAGkwtkyrhwhhdcytGCCGATAa	D	61	87	TAAAGTACTCGTTTACTTCATCTATAA	6.4	4.40E-05	-10.027
89	SO_4036	TAAAGkwtkyrhwhhdcytGCCGATAa	R	94	120	TTATGGTGTTATTTCCGCAGCCGTTAA	6.4	4.40E-05	-10.027
90	SO_3776	TAAAGkwtkyrhwhhdcytGCCGATAa	R	319	345	TGAAGGTTTTTATCTTCTAGGCGCAAT	6.3	4.70E-05	-9.962
91	SO_4018	TAAAGkwtkyrhwhhdcytGCCGATAa	R	229	255	CAAACATCTCAGTAACCTGGCCGATAT	6.3	4.70E-05	-9.962
92	SO_4019	TAAAGkwtkyrhwhhdcytGCCGATAa	D	115	141	CAAACATCTCAGTAACCTGGCCGATAT	6.3	4.70E-05	-9.962
93	SO_1701	TAAAGkwtkyrhwhhdcytGCCGATAa	D	93	119	AAAACAAAGTCAAAAAATTGTCGCTAT	6.2	5.00E-05	-9.898
94	SO_1702	TAAAGkwtkyrhwhhdcytGCCGATAa	R	211	237	AAAACAAAGTCAAAAAATTGTCGCTAT	6.2	5.00E-05	-9.898
95	fliK	TAAAGkwtkyrhwhhdcytGCCGATAa	R	40	66	TAAAGCAAGCAAATAAAATGCCAACAA	6	5.70E-05	-9.771
96	kdtA	TAAAGkwtkyrhwhhdcytGCCGATAa	D	93	119	TAAAAAAGGCTAACCAACGACCGCTAC	6	5.70E-05	-9.771
97	SO_4675	TAAAGkwtkyrhwhhdcytGCCGATAa	R	182	208	TAAAAAAGGCTAACCAACGACCGCTAC	6	5.70E-05	-9.771
98	cydD	TAAAGkwtkyrhwhhdcytGCCGATAa	D	129	155	TAAGCTACAAAACAGGCTTACCGTTAA	5.9	6.10E-05	-9.708
99	menG-2	TAAAGkwtkyrhwhhdcytGCCGATAa	D	287	313	TAAACTGTACGCAACGCGGATCAATAG	5.9	6.10E-05	-9.708
100	SO_0346	TAAAGkwtkyrhwhhdcytGCCGATAa	R	78	104	TAATGGGTGCAATTTAGTAGTCGATGA	5.9	6.10E-05	-9.708
101	SO_0588	TAAAGkwtkyrhwhhdcytGCCGATAa	R	103	129	TAAATAATGCATGGGGGGCGGCGGTAA	5.9	6.10E-05	-9.708
102	SO_1699	TAAAGkwtkyrhwhhdcytGCCGATAa	R	172	198	TAAATTTTATCTATAGATTGTGAATAA	5.9	6.10E-05	-9.708
103	SO_2226	TAAAGkwtkyrhwhhdcytGCCGATAa	R	117	143	TAAAACTTTCCCAAAACTGGCTGATAT	5.9	6.10E-05	-9.708
104	SO_2484	TAAAGkwtkyrhwhhdcytGCCGATAa	D	21	47	TAAAAAGATAATTTATCGAGACGATAA	5.9	6.10E-05	-9.708
105	SO_2805	TAAAGkwtkyrhwhhdcytGCCGATAa	R	269	295	TCAAGGCATTGTAGTGAATGACGATAA	5.9	6.10E-05	-9.708
106	SO_3781	TAAAGkwtkyrhwhhdcytGCCGATAa	R	237	263	TAAGCTACAAAACAGGCTTACCGTTAA	5.9	6.10E-05	-9.708
107	SO_4196	TAAAGkwtkyrhwhhdcytGCCGATAa	R	11	37	TAAACTGTACGCAACGCGGATCAATAG	5.9	6.10E-05	-9.708

108	SO_4340	TAAAGkwtkyrhwhhdcytGCCGATAa	R	201	227	TAAATTTAACCATGATCAGCTCGTTAA	5.9	6.10E-05	-9.708
109	pflB	TAAAGkwtkyrhwhhdcytGCCGATAa	R	181	207	TTAACGTGGATTATCGGTTATCGATAA	5.8	6.50E-05	-9.645
110	SO_0420.2	TAAAGkwtkyrhwhhdcytGCCGATAa	R	146	172	TTAAGATTATCATGGCTGCGCCGCTAG	5.8	6.50E-05	-9.645
111	SO_0500	TAAAGkwtkyrhwhhdcytGCCGATAa	R	273	299	TAAAATAAAAAAAACGGCTGACGATCA	5.8	6.50E-05	-9.645
112	SO_1942	TAAAGkwtkyrhwhhdcytGCCGATAa	D	235	261	TAAAGTGCGTTAAACTCCCGCTGATCT	5.8	6.50E-05	-9.645
113	SO_1944	TAAAGkwtkyrhwhhdcytGCCGATAa	R	60	86	TAAAGTGCGTTAAACTCCCGCTGATCT	5.8	6.50E-05	-9.645
114	SO_4665	TAAAGkwtkyrhwhhdcytGCCGATAa	D	243	269	TATTGGGTTCGAGCCCCATGCCAATAA	5.8	6.50E-05	-9.645
115	SO_2110	TAAAGkwtkyrhwhhdcytGCCGATAa	D	77	103	TATAGTTTGCGTTCTTAGTGTCCAAAA	5.7	6.90E-05	-9.582
116	SO_2228	TAAAGkwtkyrhwhhdcytGCCGATAa	D	277	303	TCAACGTTCTAATTAGCAATCAGATAA	5.7	6.90E-05	-9.582
117	SO_2844	TAAAGkwtkyrhwhhdcytGCCGATAa	R	182	208	TAACGTCGCCCAATGGCTCGCCAATAA	5.7	6.90E-05	-9.582
118	SO_4165	TAAAGkwtkyrhwhhdcytGCCGATAa	R	279	305	TAAACGTTCATCATTTAGTCACGAAAA	5.7	6.90E-05	-9.582
119	cdsA	TAAAGkwtkyrhwhhdcytGCCGATAa	R	39	65	TAATGGAATCAACCAAATTGCTGTTAT	5.6	7.30E-05	-9.52
120	cobB	TAAAGkwtkyrhwhhdcytGCCGATAa	R	55	81	TTAAGTTTTTAATTTGACCCCCTATAA	5.6	7.30E-05	-9.52
121	fur	TAAAGkwtkyrhwhhdcytGCCGATAa	D	176	202	TTAAGTTTTTAATTTGACCCCCTATAA	5.6	7.30E-05	-9.52
122	mmsB	TAAAGkwtkyrhwhhdcytGCCGATAa	D	17	43	TAAAGCAAACGCTTAGCCTGCCATTCT	5.6	7.30E-05	-9.52
123	nrdD	TAAAGkwtkyrhwhhdcytGCCGATAa	D	67	93	TTAAGTCTCTCCATTTAGCACCGCTAT	5.6	7.30E-05	-9.52
124	rbfA	TAAAGkwtkyrhwhhdcytGCCGATAa	R	35	61	TAGAGTTACCATAGCTAATGTCATTAA	5.6	7.30E-05	-9.52
125	SO_0128	TAAAGkwtkyrhwhhdcytGCCGATAa	R	48	74	TAAATTTTCACATCTGTAATACGAAAA	5.6	7.30E-05	-9.52
126	SO_0129	TAAAGkwtkyrhwhhdcytGCCGATAa	D	41	67	TAAATTTTCACATCTGTAATACGAAAA	5.6	7.30E-05	-9.52
127	SO_0774	TAAAGkwtkyrhwhhdcytGCCGATAa	R	274	300	ATAAGATGCTCCCCAGAATGCCGCTAC	5.6	7.30E-05	-9.52
128	SO_3128.2	TAAAGkwtkyrhwhhdcytGCCGATAa	D	138	164	TAGGGTATCTGTAATACCTGCTGATAT	5.6	7.30E-05	-9.52
129	SO_3131	TAAAGkwtkyrhwhhdcytGCCGATAa	R	98	124	TAGGGTATCTGTAATACCTGCTGATAT	5.6	7.30E-05	-9.52
130	SO_3573	TAAAGkwtkyrhwhhdcytGCCGATAa	D	157	183	AAAAGTTAAACAAGCAGAAACCGAAAA	5.6	7.30E-05	-9.52
131	cheV-3	TAAAGkwtkyrhwhhdcytGCCGATAa	R	18	44	AAAAGTATAGGTGCATTCTGGCGTTAC	5.5	7.80E-05	-9.458
132	flgA	TAAAGkwtkyrhwhhdcytGCCGATAa	D	162	188	AAAAGTATAGGTGCATTCTGGCGTTAC	5.5	7.80E-05	-9.458
133	SO_1210.1	TAAAGkwtkyrhwhhdcytGCCGATAa	D	58	84	TATAATTTGCGCCTTTTTTAGCGATAC	5.5	7.80E-05	-9.458
134	SO_2851	TAAAGkwtkyrhwhhdcytGCCGATAa	D	133	159	TAGTGATTCTACATAAAAGGACGATAA	5.5	7.80E-05	-9.458

135	SO_2852	TAAAGkwtkyrhwhhdcytGCCGATAa	R	109	135	TAGTGATTCTACATAAAAGGACGATAA	5.5	7.80E-05	-9.458
136	SO_4340	TAAAGkwtkyrhwhhdcytGCCGATAa	D	26	52	TACAGGATGTACCATCGGAAGCGATAG	5.5	7.80E-05	-9.458
137	SO_4341	TAAAGkwtkyrhwhhdcytGCCGATAa	R	75	101	TACAGGATGTACCATCGGAAGCGATAG	5.5	7.80E-05	-9.458
138	SO_4454	TAAAGkwtkyrhwhhdcytGCCGATAa	R	255	281	TAAACGTTTAGAAATATCGGTCGACGA	5.5	7.80E-05	-9.458
139	SO_4528	TAAAGkwtkyrhwhhdcytGCCGATAa	R	197	223	TAAAGGTTGTAGAAAGTGCAGTGATAC	5.5	7.80E-05	-9.458
140	SO_4529	TAAAGkwtkyrhwhhdcytGCCGATAa	D	100	126	TAAAGGTTGTAGAAAGTGCAGTGATAC	5.5	7.80E-05	-9.458
141	lrp	TAAAGkwtkyrhwhhdcytGCCGATAa	R	54	80	TCAGGATTATGTTGATGCTGGCGCTAA	5.4	8.30E-05	-9.397
142	SO_0026	TAAAGkwtkyrhwhhdcytGCCGATAa	D	19	45	AAAAGTATGTCACCATTGTGGCAATCA	5.4	8.30E-05	-9.397
143	SO_0027	TAAAGkwtkyrhwhhdcytGCCGATAa	R	156	182	AAAAGTATGTCACCATTGTGGCAATCA	5.4	8.30E-05	-9.397
144	SO_3047	TAAAGkwtkyrhwhhdcytGCCGATAa	D	73	99	TAAATTTACGGGCATTAATGCCGAAAA	5.4	8.30E-05	-9.397
145	SO_3371	TAAAGkwtkyrhwhhdcytGCCGATAa	D	175	201	TAAACGTGTCCAAACTCGTCGGGATAA	5.4	8.30E-05	-9.397
146	SO_4284	TAAAGkwtkyrhwhhdcytGCCGATAa	R	86	112	TCAACTTTACCAGCAACTAGCAAATAA	5.4	8.30E-05	-9.397
147	SO_1414	TAAAGkwtkyrhwhhdcytGCCGATAa	R	141	167	TAATTTATGACAACCTCCTGTTGATAA	5.3	8.80E-05	-9.336
148	SO_1415	TAAAGkwtkyrhwhhdcytGCCGATAa	D	167	193	TAATTTATGACAACCTCCTGTTGATAA	5.3	8.80E-05	-9.336
149	SO_1771.2	TAAAGkwtkyrhwhhdcytGCCGATAa	R	52	78	TACGCGAGTTAAATACCCAGCCGTTAA	5.3	8.80E-05	-9.336
150	crp	TAAAGkwtkyrhwhhdcytGCCGATAa	D	12	38	TCAAGTTAGCCTGAAATACGTCCATAA	5.2	9.40E-05	-9.274
151	phoP	TAAAGkwtkyrhwhhdcytGCCGATAa	D	20	46	TAAAGATAATGACTTTAAAGACAATAG	5.2	9.40E-05	-9.274
152	rpmE	TAAAGkwtkyrhwhhdcytGCCGATAa	R	32	58	TAGATTATCTGCGCATTTTCTCGATAA	5.2	9.40E-05	-9.274
153	SO_0083	TAAAGkwtkyrhwhhdcytGCCGATAa	R	60	86	TAAAGCGGGTCCCCAGCTCGCCGACTA	5.2	9.40E-05	-9.274
154	SO_0316	TAAAGkwtkyrhwhhdcytGCCGATAa	R	28	54	TAAACTGACAATATTTACTTTCGTTAT	5.2	9.40E-05	-9.274
155	SO_0625	TAAAGkwtkyrhwhhdcytGCCGATAa	R	344	370	TCAAGTTAGCCTGAAATACGTCCATAA	5.2	9.40E-05	-9.274
156	SO_0786	TAAAGkwtkyrhwhhdcytGCCGATAa	R	16	42	TTAATGATATTAAAATCTAGACGCTAC	5.2	9.40E-05	-9.274
157	SO_3025	TAAAGkwtkyrhwhhdcytGCCGATAa	D	294	320	TTATCTGTGCACAACATGCGCCGTTAT	5.2	9.40E-05	-9.274
158	SO_4112.1	TAAAGkwtkyrhwhhdcytGCCGATAa	D	345	371	TTAAGGTTTCTTTTAACTCGCCCCAAA	5.2	9.40E-05	-9.274
159	SO_4550	TAAAGkwtkyrhwhhdcytGCCGATAa	D	5	31	TAATCGTTATCCACCTAAAGGCAATAT	5.2	9.40E-05	-9.274
160	SO_A0002	TAAAGkwtkyrhwhhdcytGCCGATAa	R	126	152	TAAAATTATAATATTGCTCGCTGTTAT	5.2	9.40E-05	-9.274
161	rimI	TAAAGkwtkyrhwhhdcytGCCGATAa	R	7	33	TAATGTATCCAAAACACGGCGCAATAA	5.1	1.00E-04	-9.214

162	SO_1056	TAAAGkwtkyrhwhhdcytGCCGATAa	R	362	388	TAAATTCCGTTAGCAGCCTAACGTTAA	5.1	1.00E-04	-9.214
163	SO_1489	TAAAGkwtkyrhwhhdcytGCCGATAa	D	334	360	AAAACTGTGATTTATCTCAGGCGATAT	5.1	1.00E-04	-9.214
164	SO_1510	TAAAGkwtkyrhwhhdcytGCCGATAa	D	189	215	TCAAGACAATACTCTGCTCACCGAAAA	5.1	1.00E-04	-9.214
165	SO_2280	TAAAGkwtkyrhwhhdcytGCCGATAa	R	124	150	CAAATAAGCCAATCTTGGTGACGATAT	5.1	1.00E-04	-9.214
166	SO_2281	TAAAGkwtkyrhwhhdcytGCCGATAa	D	178	204	CAAATAAGCCAATCTTGGTGACGATAT	5.1	1.00E-04	-9.214
167	SO_4073	TAAAGkwtkyrhwhhdcytGCCGATAa	D	65	91	CAAAAAAGACTCAGTAACGGCCGATAA	5.1	1.00E-04	-9.214
168	SO_4075	TAAAGkwtkyrhwhhdcytGCCGATAa	R	291	317	CAAAAAAGACTCAGTAACGGCCGATAA	5.1	1.00E-04	-9.214
169	SO_A0108	TAAAGkwtkyrhwhhdcytGCCGATAa	D	330	356	TATATATAAATAATATCTCGCCGAAAA	5.1	1.00E-04	-9.214
170	SO_A0159	TAAAGkwtkyrhwhhdcytGCCGATAa	D	46	72	AAAAGGTGTTGTCATTTGTGGCGACAC	5.1	1.00E-04	-9.214
